# Effects of a WhatsApp-Assisted Health Educational Intervention for Cardiac Rehabilitation: A Randomized Controlled Clinical Trial Protocol

**DOI:** 10.3390/mps7020035

**Published:** 2024-04-19

**Authors:** Adriana Marcela Jacome-Hortua, Zully Rocio Rincon-Rueda, Diana C. Sanchez-Ramirez, Adriana Angarita-Fonseca

**Affiliations:** 1 Universidad de Santander, Facultad de Ciencias Médicas y de la Salud, Bucaramanga 680003, Colombia; ad.jacome@mail.udes.edu.co (A.M.J.-H.); zu.rincon@mail.udes.edu.co (Z.R.R.-R.); 2 Department of Respiratory Therapy, University of Manitoba, Winnipeg, MB R3E 0T6, Canada; diana.sanchez-ramirez@umanitoba.ca

**Keywords:** cardiac rehabilitation, mobile health, WhatsApp, educational intervention, cardiovascular risk

## Abstract

Although the effectiveness of cardiac rehabilitation (CR) programs in secondary prevention is well-recognized, there is a lack of studies exploring the potential of mobile health to enhance educational interventions within CR. The objective is to assess the impact of a structured WhatsApp-assisted health educational intervention, in conjunction with the usual care, compared to the usual care alone among participants enrolled in a CR program. The trial will recruit 32 participants enrolled in a CR program, who will be randomly assigned to a structured WhatsApp-assisted health educational intervention plus usual care or usual care alone group. The intervention will span 4 weeks, with assessments at baseline, 4 weeks, and 3, 6, and 12 months. The primary outcome measure is the cardiovascular risk factors knowledge score. Secondary outcomes include physical activity levels, anxiety and depression, and quality of life. Expected results include improved knowledge of cardiovascular risk factors, increased physical activity levels, and better mental health outcomes in the intervention group. Additionally, an enhancement in the overall quality of life is anticipated. These findings are expected to underscore the value of integrating mHealth with traditional CR methods, potentially shaping future approaches in chronic disease management and prevention.

## 1. Introduction

Cardiac diseases are one of the leading causes of mortality worldwide [1]. Specifically, ischemic cardiopathy was responsible for 18.6 million deaths in 2019 [1]. Cardiac rehabilitation (CR) programs, defined as multidisciplinary interventions involving exercise and education, over the last decade have emerged as a valuable secondary prevention strategy to promote lifestyle changes [2], improve cardiopulmonary fitness [3], reduce the risk of mortality [4,5], and enhance mental health [6].

The scientific literature has demonstrated that educational interventions targeting patients living with cardiovascular diseases have a positive effect on disease knowledge, behavioral changes, and health-related quality of life and decrease the recurrence of cardiac events [7,8]. Moreover, different guidelines propose specific topics that should be taught to patients with cardiomyopathies [8,9]. However, there is a lack of consensus about the most effective method for delivering this educational content effectively [10,11,12].

Mobile health (mHealth) presents itself as a versatile solution, offering an alternative to traditional in-person interventions or enhancing these visits by minimizing barriers such as distance, cost, and time [13,14,15]. This innovative approach leverages technology to provide accessible healthcare options, bridging gaps that often exist in conventional healthcare settings [13,14]. This is particularly relevant in the context of CR, where the use of technology has been rapidly expanding [12]. Among various mHealth tools, WhatsApp, a free and widely used instant messaging app, shows promise and potential in health education [16]. Its effectiveness has been demonstrated in delivering educational content on topics like tobacco consumption and cardiovascular risk to patients with heart conditions, yielding positive results in patient outcomes [17].

Studies such as those conducted by Arantes et al. [10] and Pinzon et al. [18] have highlighted the significant benefits of incorporating technology into CR programs. These benefits include improved patient adherence to CR programs, reduced anxiety, facilitated recovery processes, and delayed disease progression. Additionally, the use of educational interventions in CR has been shown to increase patients’ knowledge and awareness of risk factors and lifestyle impacts [17,19]. However, despite the growing trend in utilizing mHealth for educational purposes in CR [20], especially post-COVID-19 pandemic [21], research focusing on the Latin American region remains limited [22].

In Colombia, WhatsApp has emerged as the predominant communication platform among the adult population. Its extensive adoption is evident, with statistics showing that eight out of every ten Colombians use WhatsApp as their primary mode of communication [23]. This widespread usage positions WhatsApp as an ideal medium for effective and extensive engagement, particularly in the realm of healthcare and patient education.

Given this context, the objective of this research is to explore the potential of WhatsApp as a tool for enhancing CR programs. Specifically, this study aims to assess the impact of a structured WhatsApp-assisted health educational intervention, in conjunction with the usual care, on improving participants’ knowledge of cardiovascular risk factors, their physical activity levels, and mental health parameters such as anxiety and depression. Additionally, this study will evaluate the intervention’s effect on the overall quality of life of participants attending a CR program. These assessments will be conducted immediately following the intervention and then again 12 months later, allowing for a comparison of the long-term effects of the WhatsApp-assisted approach versus the usual care alone. This research seeks to provide valuable insights into the efficacy of integrating mobile health technology with traditional care methods in CR settings.

## 2. Experimental Design

### 2.1. Study Design

The study design is a single-blinded, two-arm, parallel, randomized controlled trial comparing the effects of a WhatsApp-assisted health educational intervention to those of the usual care on knowledge of cardiovascular risk factors, physical activity levels, and mental health in adults attending a CR program.

### 2.2. The Theoretical Basis of Our Work

This study is supported by the transtheoretical model of change (TTM) developed by Prochaska and DiClemente [24] and the andragogy that describes the adult learner [24]. The TTM can be used to determine individuals’ intention to change according to stages including precontemplation (the new behavior is not considered), contemplation (the new behavior is contemplated but not acted upon), preparation (efforts are made to prepare for adopting the new behavior), action (the initial behavior change is made), and maintenance (the new behavior is maintained over time). The andragogy [24] theory has five assumptions: somebody (1) has an independent self-concept and can direct their learning, (2) has accumulated a reservoir of life experiences that is a rich resource for learning, (3) has learning needs closely related to changing social roles, (4) is problem-centered and interested in the immediate application of knowledge, and (5) is motivated to learn by internal rather than external factors [25].

### 2.3. Study Setting

Participants will be recruited in Bucaramanga, Colombia, which is ranked the city with the ninth-largest population in the country, with 607,428 people according to the 2019 National Statistics Projections. Participants will be selected from the outpatient clinic “Profesionales de la Salud y Cia LTDA” located in a tertiary care center.

### 2.4. Participants

Eligible individuals must be over 18 years of age, possess a smartphone with a data plan or Wi-Fi access, and have proficiency in using the WhatsApp application, including basic skills to read, write, and interact with content on the platform. Additionally, they should be prescribed at least 20 CR sessions. Exclusion criteria include participation in any CR program in the past, a prior diagnosis of depression or anxiety, cognitive impairment as indicated by a Mini-Mental Test score below 25, institutionalization, severe co-morbidities contraindicating CR, or experiencing severe surgical complications such as a general stroke with severe disability.

## 3. Procedure

### 3.1. Recruitment

All patients seeking appointments at the outpatient clinic ‘Profesionales de la Salud y Cia LTDA’ for CR will undergo screening to identify those who meet these criteria. Those who qualify will be provided with detailed study information, and informed consent will be obtained before participation.

### 3.2. Randomization and Masking

Randomization will be conducted after baseline assessment. The participants will be randomized into one of the two groups: WhatsApp-assisted health educational intervention with the usual care (intervention group) or the usual care (control group). We will employ permuted block randomization for participant allocation. This approach involves generating a randomized sequence using varying block sizes to assign participants to different treatment groups. This method ensures balanced group sizes and minimizes selection bias. To maintain methodological quality and safeguard this study’s integrity, an allocation concealment strategy will be utilized involving the use of sealed opaque envelopes. These envelopes will be sequentially numbered and prepared by an individual not involved in the enrollment or assessment of participants. They will only be opened by personnel separate from the envelope preparation and assessment processes, thereby maintaining the concealment of the allocation sequence until assignment and preventing potential biases. Evaluators and data analysts will be masked to treatment allocation; however, participants will not be masked. Furthermore, the evaluator responsible for initial assessments will be blinded to group assignments, ensuring an unbiased evaluation process. The therapist administering the intervention via WhatsApp will be different from the one providing usual care, reinforcing the independence of treatment delivery from the assessment process. This structure is designed to maintain the integrity of the study by separating roles and concealing group assignments from those conducting assessments. All team members involved in this study were trained on the importance of maintaining blinding and will be regularly monitored for compliance.

### 3.3. Follow-Up

**Figure 1:**
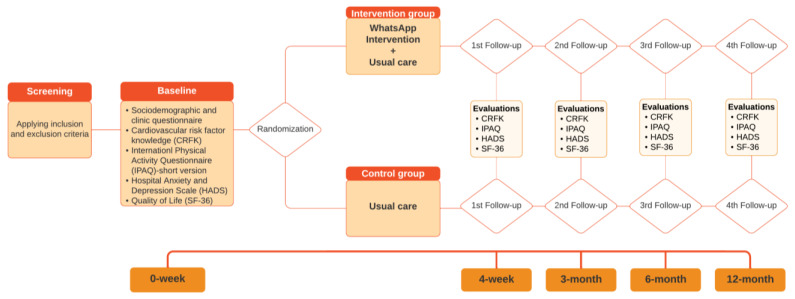
Flowchart of this study.

Post-assessments will be conducted immediately after the intervention (4 weeks), as well as at 3-, 6-, and 12-month follow-ups by telephone. See the flowchart of this study in Figure 1.

### 3.4. Interventions

The WhatsApp-assisted intervention plus usual care group (16 participants) and the usual care alone group (16 participants) will receive supervised exercise sessions and an in-person educational intervention provided by the “Profesionales de la Salud” Rehabilitation center.

#### 3.4.1. Usual Care

- Supervised exercise sessions

Participants will receive five 45-min sessions of exercise per week over 4 to 6 weeks. The number of weeks depends on the number of sessions prescribed by the cardiologist. Each session will include 5 min of warm-up and 30 min of moderate-intensity exercise on the treadmill or recumbent bike. These exercises are designed by a physiotherapist and involve a Borg scale effort perception of between 3 and 6, targeting 50% to 70% of the maximum heart rate. Additionally, the sessions will include 10 min of endurance exercise using elastic bands offering varying resistance levels, to be selected according to the participant’s progress, with each exercise performed for 8–10 repetitions. The sessions will conclude with a cooldown phase of 5 min. The sessions will be conducted in an outpatient clinic located in a tertiary care center.

- In-person educational intervention

Participants will be engaged in twice-weekly, 10 min informational sessions conducted in person by a registered physical therapist (PT) over a period of 4 to 6 weeks according to the cardiologist’s prescription. To ensure personalized attention and effective learning, each session will be conducted in small groups, limited to a maximum of 10 participants. Additionally, patients will be provided with informative flyers that offer detailed insights into their specific cardiac conditions, enhancing their understanding and engagement with their healthcare journey.

#### 3.4.2. The WhatsApp-Assisted Intervention

In addition to the usual care, the intervention group will receive a structured educational intervention delivered by daily WhatsApp messages including videos, images, and/or podcasts, explicitly designed for this study. The maximum number of participants in each WhatsApp-assisted group will be four, and, in total, there will be four groups. The WhatsApp intervention will be delivered concurrently over the same 4-week period for those with a 4-week exercise intervention and in the first 4 weeks for those with a 6-week exercise intervention. The in-person educational intervention and the WhatsApp-assisted intervention will be simultaneously conducted over the same 4-week period, ensuring a cohesive and integrated approach to patient care.

In the WhatsApp-assisted intervention, there will be a weekly theme as follows: hypertension, diabetes mellitus, smoking, and dyslipidemia. A WhatsApp message will be sent once a day for four weeks from Monday to Friday, at 8:00 AM. Participants will receive a message and an image of the physiopathology related to the weekly theme on Monday, a message and an image with relevant medication information on Tuesday, an image and a podcast about nutritional recommendations on Wednesday, a video of physical activity on Thursday, and an image with motivational phrases on Friday. In addition, every day after sending the message, a member of the research team will be available to answer questions from participants regarding the information provided.

The main functions of the WhatsApp-assisted intervention will be to enhance understanding and facilitate substantial lifestyle modifications in cardiac rehabilitation patients by providing tailored guidance on disease management, pharmacological treatments, nutrition, and physical activity through multimedia resources. The Knowing competencies will focus on educating patients about their condition and its possible repercussions, promoting awareness of heart-healthy dietary choices, emphasizing the importance of physical activity for cardiac health, and identifying risk factors linked to poor lifestyle habits. This foundational knowledge is crucial for patients to understand the why behind the recommended changes, making it easier for them to commit to these adjustments. The Being competencies concentrate on the patient’s self-awareness and reflection on their lifestyle choices. Patients are encouraged to recognize the impact of their decisions on their rehabilitation journey. This aspect underscores the importance of personal responsibility and the realization that their choices can lead to either positive or negative outcomes in their recovery process. The Doing competencies are about translating knowledge and awareness into action. Patients are expected to adhere to healthy eating recommendations, develop the ability to set practical physical activity goals, and engage in educational programs to acquire the tools needed for lifestyle modification. Through this structured approach, the WhatsApp-assisted intervention seeks not only to inform but also to transform cardiac rehabilitation patients, guiding them towards healthier lifestyle choices that support their recovery and long-term wellbeing.

### 3.5. Development of WhatsApp Messages

The development of WhatsApp messages will be a critical component. The authors will follow the steps outlined by the Pan American Health Organization (PAHO) for the creation of edu-communicative material [26]. This process will ensure that the educational content is not only informative but also engaging and tailored to the specific needs of the participants.

#### 3.5.1. Defining the Health Situation

Initially, the team will define the current health situation, focusing on the prevalence and impact of cardiovascular diseases. This step will involve a thorough analysis of the latest research and statistics to ensure that the content is relevant and up-to-date.

#### 3.5.2. Identifying Educational Needs

The next step will involve identifying the educational needs of the participants. This will be achieved through interviews with potential participants and healthcare professionals. The goal is to understand the gaps in knowledge and misconceptions about cardiovascular health, rehabilitation, and lifestyle changes.

#### 3.5.3. Strategic Definition of Content

Once the educational needs are identified, the team will strategically define the content to be included in the WhatsApp messages. This content will be designed to address the identified needs in an interactive and engaging manner.

#### 3.5.4. Strategic Plan for Production

The team will use free software tools such as Canva, Powtoon, Movie Maker, and PowerPoint to create a total of 27 pieces, including images, pop quizzes, and videos. These tools will enable the creation of visually appealing and easy-to-understand content.

#### 3.5.5. Design and Validation

Once the initial designs are complete, they will undergo a validation process. This process will involve presenting the material to seven health science experts and six users from a program to gather feedback. Their insights will be invaluable in refining the content to ensure it is effective and resonates with the audience.

### 3.6. Adherence to the Intervention

Adherence to the supervised exercise sessions and the in-person educational intervention will be monitored by an attendance signature. In addition, a member of the research team will check the WhatsApp read receipts through the WhatsApp option “read by” every day at 8 PM from Monday to Friday.

### 3.7. Providers

The usual care will not be provided by the research group, but will be delivered by healthcare providers at the rehabilitation center. The WhatsApp intervention will be delivered by two physiotherapists. One of them is specialized in CR, completed a master’s in education, and has 12 years of clinical experience in CR. The second physiotherapist is specialized in neurorehabilitation, completed a master’s in physiotherapy, and has 14 years of clinical experience in CR.

### 3.8. Primary Outcome

Cardiovascular Risk Factors Knowledge (CRFK) Score: Specifically designed and validated for this study, the knowledge questionnaire (Supplementary Materials) comprises 18 items across four disease-based dimensions: hypertension (4 items), diabetes mellitus (5 items), smoking (4 items), and dyslipidemia (5 items). Each item, structured as a statement, invites respondents to self-assess their knowledge using a five-point Likert scale, ranging from ‘No Knowledge’ (1) to ‘Extensive Knowledge’ (5). The knowledge score for each disease dimension is the average of its corresponding items. Additionally, through these 18 questions, four thematic dimensions are covered: pathophysiology (4 items), pharmacology (4 items), nutrition (6 items), and physical activity (4 items) (see Supplementary Materials). Each thematic dimension is scored from 1 to 5, where a higher score reflects more extensive understanding.

### 3.9. Secondary Outcomes

#### 3.9.1. Physical Activity Levels

These will be assessed by the International Physical Activity Questionnaire (IPAQ) [27], which has shown good test–retest reliability, achieving an intraclass correlation coefficient of 0.70 in Colombian adults [28]. The IPAQ provides detailed insights into the time spent walking or engaging in moderate- or vigorous-intensity activities as well as in sedentary behaviors.

#### 3.9.2. Anxiety and Depression Scale

The Hospital Anxiety and Depression Scale (HADS) [29] will be used for assessment. The reliability of the HADS was confirmed in a primary care setting in Colombia, with Cronbach’s α at 0.85 and McDonald’s ω at 0.82; confirmatory factor analysis supported a satisfactory two-factor structure [30]. This self-administered scale, commonly used in non-psychiatric hospital settings and primary care, consists of 14 items. Each item offers four response options, scored from 0 to 3. The total score ranges from 0 to 21, with 0 to 7 indicating a normal range, while scores from 8 to 21 are indicative of borderline or clinical concerns about anxiety or depression.

#### 3.9.3. Quality-of-Life Score

The Short Form-36 Health Survey (SF-36) will be employed for this assessment. In Colombia, it has been validated in healthy individuals and those with chronic pain, diabetes, or depression, showing a test–retest reliability of above 0.70 [31] and exhibiting a robust inter-rater reproducibility with an intraclass correlation coefficient of 0.80 [31]. The SF-36 questionnaire consists of 36 items evaluating both positive and negative health states across eight domains: physical functioning, role–physical, bodily pain, general health, vitality, social functioning, role–emotional, and mental health [32].

### 3.10. Sample Size Calculation

The sample size was calculated considering the main outcome and a test for a comparison of means. This was based on the mean and standard deviation obtained in a previous study carried out by Tang et al. (2018) [19], a power of 80%, a significance level of 5%, and a 1: 1 ratio. Additionally, we doubled the sample size, anticipating possible losses to follow-up. The calculation was performed using the statistical software STATA 16.1. The result obtained was 16 participants per study group.

### 3.11. Statistical Analysis

Descriptive statistics such as means and standard deviations or medians and interquartile ranges for continuous variables and frequency tables for categorical variables will be used.

The bivariate analysis will be carried out to evaluate the differences in the variables measured within each group and between the study groups using parametric or non-parametric tests.

To evaluate the changes through follow-up, a repeated-measures analysis of variance will be applied if data follow a normal distribution, or a non-parametric variance analysis (ATS) if data do not follow a normal distribution [33]. Multivariable generalized estimating equation (GEE) models [34] will be applied to determine the effects of group, time, and the interaction between group and time on physical activity levels.

### 3.12. Ethical Considerations

The authors declare that the procedures will follow the ethical standards of the Committee of Responsible Human Experimentation, the World Medical Association, and the Declaration of Helsinki. The ethical principles of confidentiality, charity, non-malfunction, autonomy, and justice will be respected. The authors will obtain the informed consent of the recruited participants. This research was approved by the Universidad de Santander—Bucaramanga Research Ethics Board (Number: 023, 8 June 2021) and registered in the Australian New Zealand Clinical Trials Registry (ACTRN12622001446752).

### 3.13. Handling and Storage of Data

The data will be collected, processed, and stored in accordance with the policy for data handling as described in Law 1581 of 2012 of the Republic of Colombia [35], and their use will be exclusively for academic purposes. Electronic spreadsheets will be utilized for data entry, and Microsoft OneDrive will serve as the storage medium on a secure server provided by the university. All data will be treated confidentially and anonymized for the purpose of analysis. During and after this study, physical copies of the data and related documents will be securely kept in a locked file cabinet for 7 years.

## 4. Expected Results

This study aims to assess the effects of a structured WhatsApp-assisted health educational intervention on the knowledge of cardiovascular risk factors, physical activity levels, and mental health, including anxiety and depression, as well as the overall quality of life among participants in a CR program. Recognizing the proven effectiveness of CR in various clinical scenarios for patients with cardiovascular diseases, these programs will need to include health educational processes tailored to the participants’ needs.

Building on this foundation, our protocol will seek to evaluate the impact of CR by enhancing the educational component through technology. We will be utilizing WhatsApp, a free, widely used platform, to deliver this intervention. While the use of social networks for educational purposes is increasingly reported in the literature, publications in the Latin American context remain scarce. This presents an opportunity to showcase a low-cost community intervention strategy.

The educational strategy, designed to be delivered over four weeks, will align with the characteristics of CR programs in Colombia. These programs vary in frequency and duration but typically span at least a month. Our digital educational resources, animated and easy to understand, will have been validated by experts and the target audience. They will aim to provide clear, precise, relevant, truthful, timely, and updated information, covering the four most prevalent cardiovascular risk factors in Colombia: hypertension, diabetes mellitus, hypercholesterolemia, and smoking. The content will encompass physiology, medication, diet, physical activity, and emotional management.

We expect a notable improvement in participants’ understanding of cardiovascular risk factors due to the interactive and personalized nature of the WhatsApp-assisted educational intervention. The intervention group will likely show a significant increase in their cardiovascular risk factor knowledge score compared to the control group. Additionally, an increase in physical activity levels among participants receiving the intervention is expected. The daily engagement through WhatsApp, with its motivational messages and informative content, will likely encourage participants to adopt more active lifestyles, measurable through the IPAQ scores.

Another key expectation is the positive impact of the intervention on mental health. Although we acknowledge that increased awareness could potentially lead to anxiety if not properly managed, the structure of our intervention is designed to provide support and reassurance, thereby mitigating negative emotional impacts and promoting mental wellbeing. Therefore, we predict a reduction in anxiety and depression scores, as measured by the HADS, especially in the intervention group. The supportive nature of the intervention, coupled with regular information dissemination about cardiovascular health, is expected to alleviate participants’ stress and anxiety. Research has demonstrated that in anxious individuals, having more knowledge about their condition and prognosis can help modulate their behaviors in a positive way. For instance, de la Maza et al. (2015) [36] have shown that an educational program for parents of children with cancer not only increased their knowledge about the disease but also reduced their anxiety levels. Similarly, Çankaya and Şimşek (2021) [37] and Khalilzadeh Ganjalikhani et al. (2019) [38] support the notion that health education can effectively reduce anxiety, improve self-efficacy, and enhance the overall quality of life. Furthermore, an improvement in the quality of life, as reflected in the SF-36 scores, is also anticipated. The comprehensive educational content, which includes aspects like diet, exercise, and medication management, is likely to contribute to this overall enhancement in life quality.

An important aspect of this study will be the evaluation of the intervention’s sustained impact over time. We expect that the improvements in knowledge, physical activity, mental health, and quality of life will not only be evident immediately after the intervention but will also persist during the follow-up assessments at 3, 6, and 12 months. High adherence to the intervention is also anticipated, given the convenience and familiarity of the WhatsApp platform. The ease of receiving and interacting with educational content through a widely used app is expected to foster consistent participant engagement.

In conclusion, this study is poised to offer significant insights into the effectiveness of using a popular digital platform for health education in CR. The anticipated results are expected to align with the evolving landscape of digital health interventions in chronic disease management and prevention.

## Data Availability

As this document is a protocol, there are currently no data available for sharing. Data and educational material will become accessible in accordance with this study’s progression and subsequent data collection phases.

## Supplementary Materials

The following supporting information can be downloaded at https://www.mdpi.com/article/10.3390/mps7020035/s1, Supplementary Materials. Cardiovascular risk factors knowledge questionnaire.
