# Supplementary material for: Effects of a WhatsApp-Assisted Health Educational Intervention for Cardiac Rehabilitation: A Randomized Controlled Clinical Trial Protocol
Source: Methods Protoc. 2024 Apr 19;7(2):35. doi: 10.3390/mps7020035 (PMC11053574; doi:10.3390/mps7020035)
Supplement: Supplementary file 1 [file mps-07-00035-s001.zip › mps-2940979-supplementary.pdf]

Supplementary Materials. Cardiovascular risk factors knowledge questionnaire.

Unique Participant Code

Questionnaire:

Evaluation of the knowledge about healthy habits in people attending a cardiac rehabilitation program

Name:____ Telephone Number:______________________________

Rate from 1 to 5 the knowledge you think you have about healthy habits, considering that 1 is no knowledge and 5 is extensive knowledge.

Hypertension

	Item	1 No Knowledge	2	3	4	5 Extensive Knowledge
H1	I am aware about the damage that hypertension can cause to different organs like the brain, eyes, kidneys, and heart.					
H2	I am aware of the side effects (undesirable effects) that may occur when taking the medications prescribed by the doctor for the management of hypertension.					
H3	I know the foods that can increase my blood pressure due to their high salt concentration.					
H4	I recognize the benefits of regular physical exercise on my blood pressure.					

Diabetes Mellitus

	Item	1 No Knowledge	2	3	4	5 Extensive Knowledge
DM1	I have knowledge about the damage that type 2 diabetes mellitus can cause to different organs like the brain, eyes, kidneys, heart, and legs.					
DM2	I recognize the side effects that can be felt with the medications taken for the management of diabetes.					
DM3	I am aware of the foods that can increase daily blood sugar levels (glycemia), as well as the ideal ranges in which I should maintain my daily glycemia.					
DM4	I know what glycated hemoglobin is and the values in which it is considered adequate.					
DM5	I identify the benefits that regular physical exercise can generate for blood sugar control.					

Smoking

	Item	1 No Knowledge	2	3	4	5 Extensive Knowledge
S1	I have knowledge about the damage that tobacco consumption can cause to different organs like eyes, teeth, heart, and lungs.					x
S2	I identify different strategies that exist to help reduce tobacco consumption, such as: nicotine gum, nicotine patches, and therapy with psychology.					
S3	I recognize the types of foods and the proportion they should have in a healthy plate.					
S4	I understand the role of physical exercise in effectively aiding the control and treatment of smoking-related health issues.					

Dyslipidemia

	Item	1<br>No Knowledge	2	3	4	5<br>Extensive Knowledge
D1	I possess a clear understanding of dyslipidemia, including the distinctions between high-density lipoprotein (HDL) or 'good' cholesterol and low-density lipoprotein (LDL) or 'bad' cholesterol.					
D2	I recognize the side effects that can be felt with the medications prescribed by the doctor for the control of high cholesterol.					
D3	I am aware of the foods that should not be consumed and those that should be to prevent high cholesterol.					
D4	I understand the beneficial impact of physical exercise in regulating cholesterol levels, specifically by increasing high-density lipoprotein (HDL, the 'good' cholesterol) and decreasing low-density lipoprotein (LDL, the 'bad' cholesterol) and triglycerides."					
D5	I am able to read and understand the nutritional label of the product I buy in the grocery sotore.					

Score calculation by dimension:

Hypertension Knowledge: average of H1, H2, H3, H4
Diabetes Mellitus Knowledge: average of DM1, DM2, DM3, DM4 and DM5
Smoking Knowledge: average of S1, S2, S3 and S4
Dyslipidemia Knowledge: D1, D2, D3, D4, D5

Pathophsyiology Knowledge: average of H1, DM1, S1, D1,
Pharmacology knowledge: average of H2, DM2, S2, D2
Nutritional Knowledge: average of H3, DM3, DM4, S3, D3, D5
Physical activity knowledge: average of H4, DM5, S4, D4
